# Supplementary material for: Leaf morpho-physiological traits of Populus sibirica and Ulmus pumila in different irrigation regimes and fertilizer types
Source: PeerJ. 2023 Sep 29;11:e16107. doi: 10.7717/peerj.16107 (PMC10544310; doi:10.7717/peerj.16107)
Supplement: Supplemental Information 3 [file peerj-11-16107-s003.docx]

Table S2 P values estimated by one-way analysis of variance (ANOVA) for leaf physiological across treatments. Showing sources of variance, degrees of freedom for numerator (DF) and F ratios (F value) and their probabilities (Pr) treatment. P value in bold font indicates non-significant at *α* = 0.05.

| Source | Species | DF | 2021 | | 2022 | |
| --- | --- | --- | --- | --- | --- | --- |
|  |  |  | F Value | Pr>F | F Value | Pr>F |
| *Chlorophyll content* | *P. sibirica* | 9 | 0.75 | 0.6797 | 1.56 | 0.1732 |
|  | *U. pumila* | 11 | 2.26 | 0.0648 | 1.09 | 0.4144 |
| *Stomatal conductance* | *P. sibirica* | 9 | 2.66 | 0.0056 | 3.28 | 0.0009 |
|  | *U. pumila* | 11 | 5.48 | <.0001 | 22.69 | <.0001 |
| F_0_ | *P. sibirica* | 9 | 3.02 | 0.0038 | 2.59 | 0.0113 |
|  | *U. pumila* | 11 | 4.79 | <.0001 | 3.19 | 0.0010 |
| F_m_ | *P. sibirica* | 9 | 2.53 | 0.0135 | 6.51 | <.0001 |
|  | *U. pumila* | 11 | 1.51 | 0.1434 | 2.39 | 0.0116 |
| F_v_ | *P. sibirica* | 9 | 3.18 | 0.0026 | 7.95 | <.0001 |
|  | *U. pumila* | 11 | 2.23 | 0.0194 | 2.77 | 0.0038 |
| F_v_/F_m_ | *P. sibirica* | 9 | 3.87 | 0.0004 | 7.98 | <.0001 |
|  | *U. pumila* | 11 | 4.20 | <.0001 | 3.63 | 0.0003 |
| PI_ABS_ | *P. sibirica* | 9 | 5.02 | <.0001 | 3.82 | 0.0005 |
|  | *U. pumila* | 11 | 7.35 | <.0001 | 5.94 | <.0001 |
| ψ_p_ | *P. sibirica* | 9 | 9.01 | <.0001 | 4.09 | 0.0053 |
|  | *U. pumila* | 11 | 6.35 | <.0001 | 5.41 | 0.0003 |
| ψ_m_ | *P. sibirica* | 9 | 9.72 | <.0001 | 2.77 | 0.0279 |
|  | *U. pumila* | 11 | 4.50 | 0.0010 | 3.81 | 0.0030 |
