# Supplementary material for: Leaf morpho-physiological traits of Populus sibirica and Ulmus pumila in different irrigation regimes and fertilizer types
Source: PeerJ. 2023 Sep 29;11:e16107. doi: 10.7717/peerj.16107 (PMC10544310; doi:10.7717/peerj.16107)
Supplement: Supplemental Information 4 — Showing sources of variance, degrees of freedom for numerator (DF) and F ratios (F value) and their probabilities (Pr) treatment. P value in bold font indicates non-significant at α = 0.05. [file peerj-11-16107-s004.docx]

**Supplemental files**

Table S1 P values estimated by one-way analysis of variance (ANOVA) for leaf morphological traits across treatments. Showing sources of variance, degrees of freedom for numerator (DF) and F ratios (F value) and their probabilities (Pr) treatment. P value in bold font indicates non-significant at *α* = 0.05.

| Source | Species | DF | 2021 | | | 2022 | |
| --- | --- | --- | --- | --- | --- | --- | --- |
|  |  |  | F Value | | Pr>F | F Value | Pr>F |
| Leaf area | *P. sibirica* |  | 2.96 | **0.0017** | | 5.72 | **<.0001** |
|  | *U. pumila* | 11 | 17.16 | **<.0001** | | 6.83 | **<.0001** |
| SLA | *P. sibirica* |  | 1.47 | 0.1523 | | 4.16 | **0.0001** |
|  | *U. pumila* | 11 | 18.16 | **<.0001** | | 4.30 | **<.0001** |
