# Supplementary material for: Leaf morpho-physiological traits of Populus sibirica and Ulmus pumila in different irrigation regimes and fertilizer types
Source: PeerJ. 2023 Sep 29;11:e16107. doi: 10.7717/peerj.16107 (PMC10544310; doi:10.7717/peerj.16107)
Supplement: Supplemental Information 5 — Showing sources of variance, degrees of freedom for numerator (DF) and F ratios (F value) and their probabilities (Pr) treatment. P value in bold font indicates non-significant at α= 0.05. [file peerj-11-16107-s005.docx]

**Supplemental files**

Table S2 P values estimated by three-way analysis of variance (ANOVA) for leaf morphological traits across treatments. Showing sources of variance, degrees of freedom for numerator (DF) and F ratios (F value) and their probabilities (Pr) treatment. P value in bold font indicates non-significant at *α* = 0.05.

| Species | Source | DF | Leaf area | | SLA | |
| --- | --- | --- | --- | --- | --- | --- |
|  |  |  | F Value | Pr > F | F Value | Pr > F |
| *P. sibirica* | year | 1 | 434.24 | <.0001 | 0.24 | 0.6281 |
|  | Irrigation | 3 | 7.58 | <.0001 | 1.20 | 0.3111 |
|  | fertilization | 2 | 3.84 | 0.0230 | 7.69 | 0.0006 |
|  | year*irrigation | 3 | 9.59 | <.0001 | 4.40 | 0.0050 |
|  | year*fertilization | 2 | 7.37 | 0.0008 | 0.59 | 0.5562 |
|  | irrigation*fertilization | 6 | 0.70 | 0.6469 | 2.54 | 0.0213 |
|  | year*irrigation*fertilization | 4 | 6.13 | 0.0001 | 0.90 | 0.4647 |
| *U. pumila* | year | 1 | 210.47 | <.0001 | 17.41 | <.0001 |
|  | Irrigation | 3 | 11.91 | <.0001 | 16.55 | <.0001 |
|  | fertilization | 2 | 2.85 | 0.0596 | 6.27 | 0.0022 |
|  | year*irrigation | 3 | 0.28 | 0.8433 | 3.65 | 0.0132 |
|  | year*fertilization | 2 | 1.87 | 0.1554 | 0.90 | 0.4061 |
|  | irrigation*fertilization | 6 | 27.16 | <.0001 | 5.54 | <.0001 |
|  | year*irrigation*fertilization | 6 | 11.53 | <.0001 | 2.78 | 0.0123 |
