# Supplementary material for: Leaf morpho-physiological traits of Populus sibirica and Ulmus pumila in different irrigation regimes and fertilizer types
Source: PeerJ. 2023 Sep 29;11:e16107. doi: 10.7717/peerj.16107 (PMC10544310; doi:10.7717/peerj.16107)
Supplement: Supplemental Information 7 — Showing sources of variance, degrees of freedom for numerator (DF) and F ratios (F value) and their probabilities (Pr) treatment. P value in bold font indicates non-significant at α = 0.05. [file peerj-11-16107-s007.docx]

Table S4 P values estimated by three-way analysis of variance (ANOVA) for chlorophyll content and stomatal conductance across treatments. Showing sources of variance, degrees of freedom for numerator (DF) and F ratios (F value) and their probabilities (Pr) treatment. P value in bold font indicates non-significant at *α* = 0.05.

| Species | Source | DF | *Chlorophyll content* | | *Stomatal conductance* | |
| --- | --- | --- | --- | --- | --- | --- |
|  |  |  | F Value | Pr > F | F Value | Pr > F |
| *P. sibirica* | year | 1 | 0.27 | 0.6067 | 1.06 | 0.3042 |
|  | Irrigation | 3 | 0.62 | 0.6092 | 24.67 | <.0001 |
|  | fertilization | 2 | 3.36 | 0.0449 | 47.74 | <.0001 |
|  | year*irrigation | 3 | 2.93 | 0.0453 | 2.73 | 0.0453 |
|  | year*fertilization | 2 | 0.55 | 0.5788 | 3.56 | 0.0304 |
|  | irrigation*fertilization | 4 | 1.61 | 0.1917 | 7.45 | <.0001 |
|  | year*irrigation*fertilization | 4 | 0.26 | 0.9011 | 5.76 | <.0001 |
| *U. pumila* | year | 1 | 15.85 | 0.0002 | 11.04 | 0.0011 |
|  | Irrigation | 3 | 0.56 | 0.6432 | 4.17 | 0.0071 |
|  | fertilization | 2 | 1.81 | 0.1753 | 6.11 | 0.0028 |
|  | year*irrigation | 3 | 1.31 | 0.2835 | 0.46 | 0.7122 |
|  | year*fertilization | 2 | 0.62 | 0.5447 | 12.45 | <.0001 |
|  | irrigation*fertilization | 6 | 1.2 | 0.3218 | 1.16 | 0.3309 |
|  | year*irrigation*fertilization | 6 | 1.34 | 0.2584 | 2.83 | 0.0264 |
