# Supplementary material for: Leaf morpho-physiological traits of Populus sibirica and Ulmus pumila in different irrigation regimes and fertilizer types
Source: PeerJ. 2023 Sep 29;11:e16107. doi: 10.7717/peerj.16107 (PMC10544310; doi:10.7717/peerj.16107)
Supplement: Supplemental Information 8 — Showing sources of variance, degrees of freedom for numerator (DF) and F ratios (F value) and their probabilities (Pr) treatment. P value in bold font indicates non-significant at α = 0.05. [file peerj-11-16107-s008.docx]

Table S5 P values estimated by three-way analysis of variance (ANOVA) for chlorophyll fluorescents across treatments. Showing sources of variance, degrees of freedom for numerator (DF) and F ratios (F value) and their probabilities (Pr) treatment. P value in bold font indicates non-significant at *α* = 0.05.

| Species | Source | DF | F_0_ | | F_m_ | | F_v_ | | F_v_/F_m_ | | PI_ABS_ | |
| --- | --- | --- | --- | --- | --- | --- | --- | --- | --- | --- | --- | --- |
|  |  |  | F Value | Pr > F | F Value | Pr > F | F Value | Pr > F | F Value | Pr > F | F Value | Pr > F |
| *P. sibirica* | year | 1 | 0.26 | 0.6079 | 73.89 | <.0001 | 74.81 | <.0001 | 32.58 | <.0001 | 37.61 | <.0001 |
|  | Irrigation | 3 | 5.16 | 0.0020 | 2.84 | 0.0400 | 4.55 | 0.0044 | 9.14 | <.0001 | 5.83 | 0.0008 |
|  | fertilization | 2 | 8.54 | 0.0003 | 2.94 | 0.0557 | 3.38 | 0.0366 | 6.11 | 0.0028 | 13.02 | <.0001 |
|  | year*irrigation | 3 | 0.19 | 0.9006 | 1.12 | 0.3420 | 1.22 | 0.3059 | 1.55 | 0.2040 | 1.24 | 0.2972 |
|  | year*fertilization | 2 | 2.64 | 0.0742 | 22.99 | <.0001 | 27.27 | <.0001 | 23.21 | <.0001 | 18.67 | <.0001 |
|  | irrigation*fertilization | 4 | 1.85 | 0.1227 | 1.54 | 0.1923 | 1.26 | 0.2869 | 0.65 | 0.6303 | 1.32 | 0.2653 |
|  | year*irrigation*fertilization | 4 | 1.40 | 0.2368 | 0.68 | 0.6067 | 0.30 | 0.8753 | 0.45 | 0.7413 | 0.29 | 0.8830 |
| *U. pumila* | year | 1 | 32.27 | <.0001 | 4.43 | 0.0366 | 1.00 | 0.3193 | 1.99 | 0.1605 | 3.75 | 0.0545 |
|  | Irrigation | 3 | 3.80 | 0.0113 | 2.41 | 0.0685 | 3.33 | 0.0207 | 4.96 | 0.0025 | 4.45 | 0.0048 |
|  | fertilization | 2 | 1.49 | 0.2289 | 2.34 | 0.0993 | 2.13 | 0.1213 | 1.46 | 0.2357 | 1.20 | 0.3036 |
|  | year*irrigation | 3 | 0.60 | 0.6135 | 1.34 | 0.2621 | 1.07 | 0.3629 | 0.43 | 0.7302 | 1.31 | 0.2716 |
|  | year*fertilization | 2 | 10.72 | <.0001 | 6.59 | 0.0017 | 9.06 | 0.0002 | 14.89 | <.0001 | 10.74 | <.0001 |
|  | irrigation*fertilization | 6 | 7.65 | <.0001 | 2.03 | 0.0641 | 2.93 | 0.0094 | 5.52 | <.0001 | 6.05 | <.0001 |
|  | year*irrigation*fertilization | 6 | 0.07 | 0.9987 | 0.82 | 0.5567 | 0.72 | 0.6305 | 0.88 | 0.5140 | 0.03 | 0.9999 |
