# Supplementary figures and images for: Leaf morpho-physiological traits of Populus sibirica and Ulmus pumila in different irrigation regimes and fertilizer types
Source: PeerJ. 2023 Sep 29;11:e16107. doi: 10.7717/peerj.16107 (PMC10544310; doi:10.7717/peerj.16107)

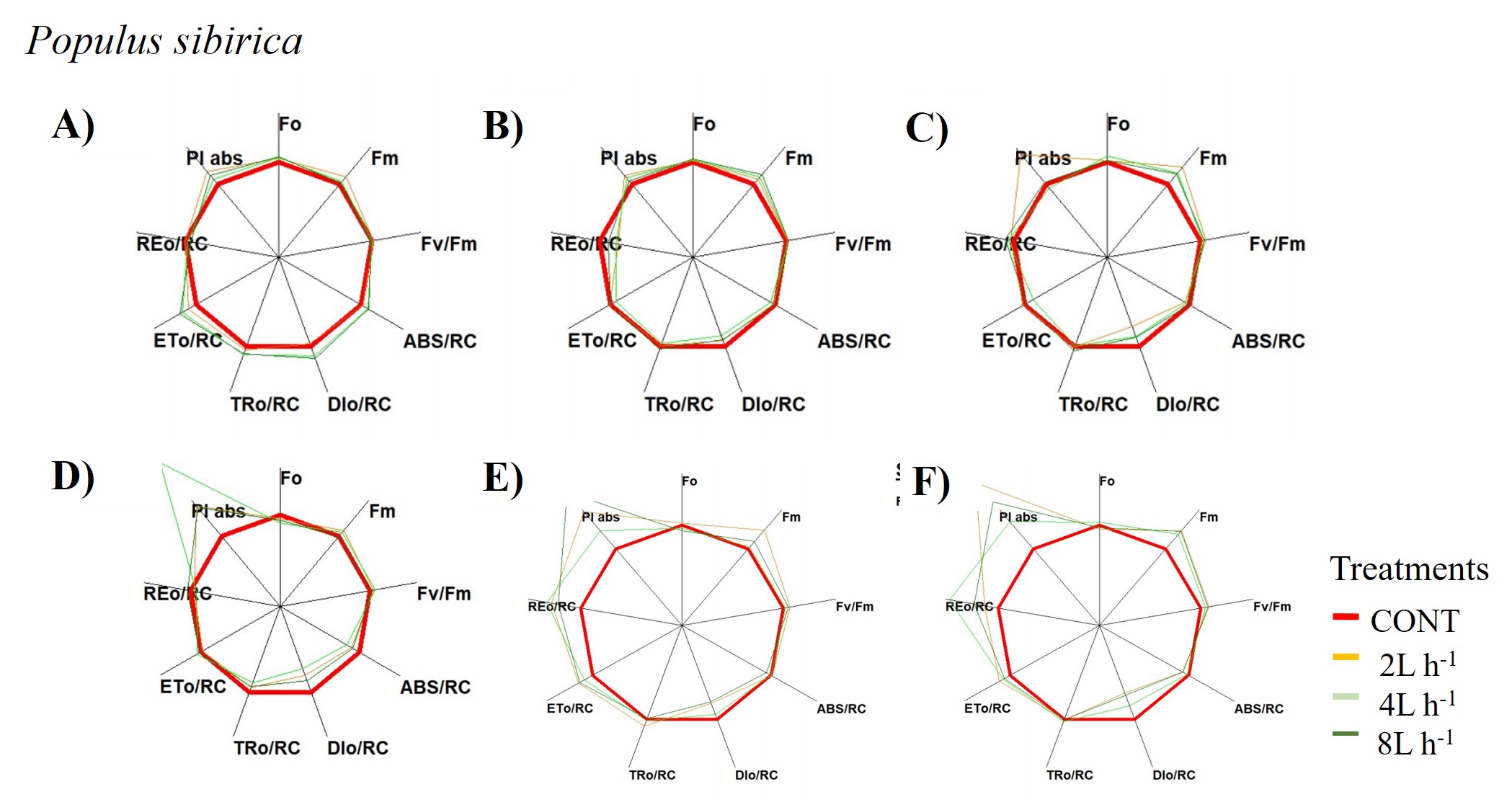

Supplement: Supplemental Information 9 — (A) only water treatments (B) water+NPK treatments (C) water+COMP treatments in July, 2021 and (D) only water treatments (E) water+NPK treatments (F) water+COMP in July, 2022. [file peerj-11-16107-s009.jpg]

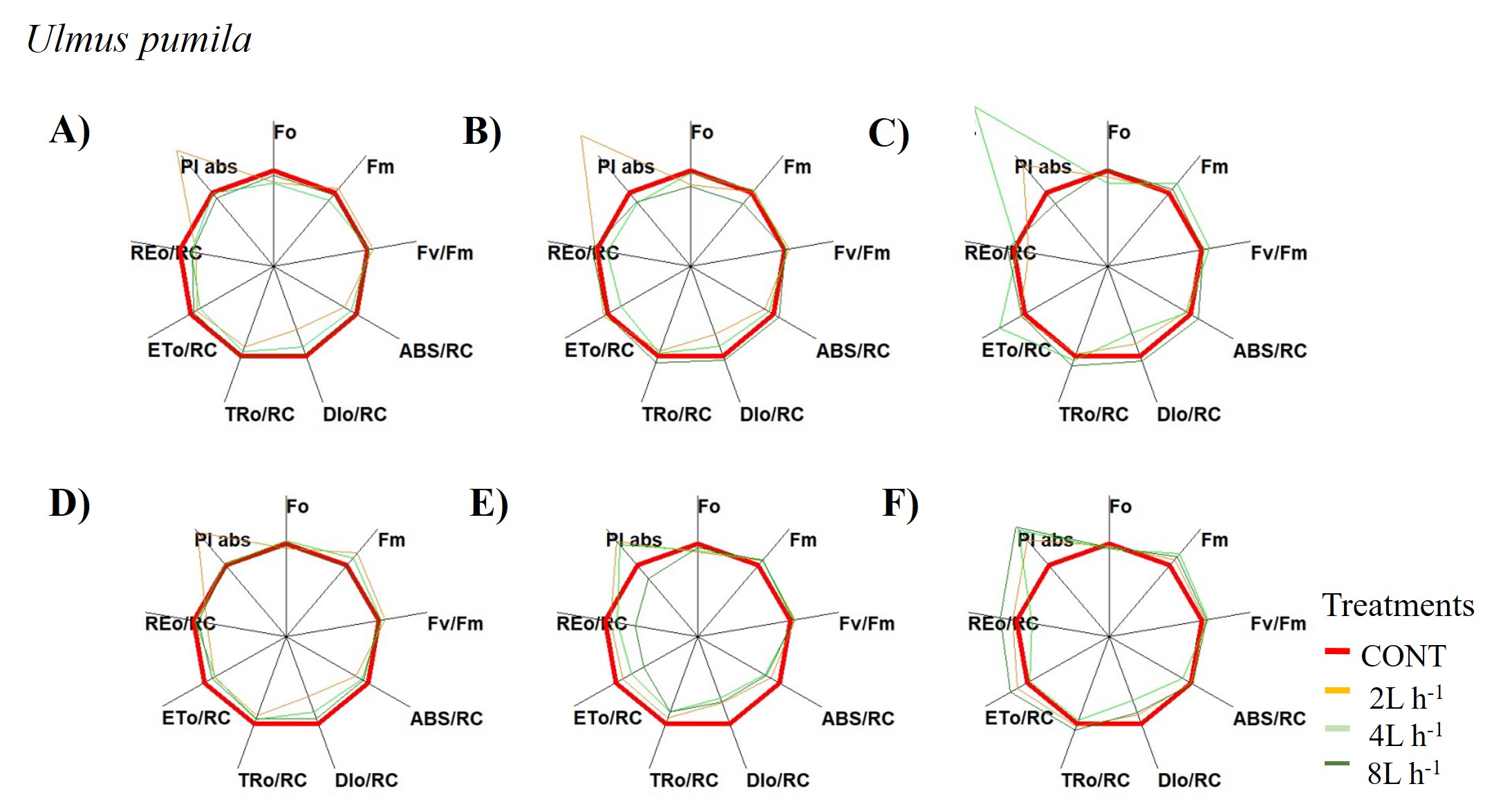

Supplement: Supplemental Information 10 — A) only water treatments B) water+NPK treatments C) water+COMP treatments in July, 2021 and D) only water treatments E) water+NPK treatments F) water+COMP in July, 2022. [file peerj-11-16107-s010.jpg]
